# Deciphering the secrets of tumor-initiating cells in pancreatic ductal adenocarcinoma microenvironment: mechanisms and therapeutic opportunities

**DOI:** 10.3389/fimmu.2025.1614707

**Published:** 2025-08-13

**Authors:** Jun Zhou, Jiajun Li, Haoyi Lu, Yanggang Hong

**Affiliations:** ^1^ Wenzhou Medical University, Wenzhou, Zhejiang, China; ^2^ State Key Laboratory of Systems Medicine for Cancer, Shanghai Cancer Institute and Department of Liver Surgery, Renji Hospital, Shanghai Jiao Tong University School of Medicine, Shanghai, China

**Keywords:** pancreatic ductal adenocarcinoma, tumor-initiating cell, cancer stem cell, tumor microenvironment, immune evasion

## Abstract

Pancreatic ductal adenocarcinoma (PDAC) is a highly aggressive malignancy characterized by poor prognosis, strong resistance to therapy, and a dense immunosuppressive tumor microenvironment (TME). A small subset of cells known as cancer stem cells (CSCs), or tumor-initiating cells (TICs), are increasingly recognized as key contributors to tumor initiation, metastasis, immune evasion, and treatment failure. These cells are defined by their self-renewal capacity, plasticity, and resistance to chemotherapeutic and targeted therapies. Pancreatic cancer stem cells (PaCSCs) are maintained by specific surface markers (CD44, CD133, EpCAM, ALDH1A1) and regulated by stemness-associated signaling pathways such as Wnt/β-catenin, Notch, Hedgehog, and TGF-β. Their survival is further enhanced by metabolic reprogramming, including shifts between glycolysis and oxidative phosphorylation and the activation of ROS-detoxifying enzymes. Importantly, PaCSCs reside in specialized niches formed by hypoxia, cancer-associated fibroblasts (CAFs), tumor-associated macrophages (TAMs), and extracellular matrix (ECM) components that together shield them from immune clearance and promote therapeutic resistance. This review outlines the molecular features and functional roles of PaCSCs, their interaction with the TME, and recent advances in targeting this CSC-stroma network. Promising therapeutic strategies, such as CAR-T/NK cell therapies, epigenetic inhibitors, and combination regimens with checkpoint blockade or stromal modulators, are discussed in the context of ongoing clinical trials. Targeting both CSCs and their supportive microenvironment is emerging as a necessary strategy to overcome resistance and improve clinical outcomes in PDAC.

## 1 Introduction

Pancreatic ductal adenocarcinoma (PDAC) is one of the most lethal malignancies, characterized by late diagnosis, rapid progression, and limited therapeutic efficacy (1). Despite accounting for only about 3% of all cancers, PDAC is the fourth leading cause of cancer-related deaths worldwide, with a 5-year survival rate below 15% (2). The aggressive nature of PDAC arises from its early metastasis, tumor immune environment, and profound resistance to conventional treatments (3). Although combinations such as FOLFIRINOX or nab-paclitaxel with gemcitabine have prolonged overall survival (OS) of patients, durable responses remain rare (4). These clinical challenges reflect the urgent need to better understand the cellular and molecular mechanisms that drive PDAC aggressiveness and therapy failure.

A major contributor to PDAC’s dismal prognosis is its intrinsic and acquired therapeutic resistance (4). Mounting evidence suggests that a rare subpopulation of cells known as cancer stem cells (CSCs), also referred to as tumor-initiating cells (TICs), plays a central role in orchestrating this resistance (5, 6). These cells exhibit self-renewal capacity, tumor-initiating potential, and resilience under stressful conditions, including nutrient-deprived and hypoxic microenvironments (7). Studies have shown that gemcitabine treatment enriches for CD133^+^ and CD24^+^ pancreatic cancer stem cells (PaCSCs), which display enhanced sphere-forming ability, drug efflux capacity, and tumorigenicity (8–10). Moreover, CSCs are protected by a supportive niche that includes hypoxia, immunosuppressive immune cells, and extracellular matrix (ECM) barriers, all of which hinder drug penetration and immune-mediated clearance (5, 11, 12). The failure of single-agent targeted therapies, such as Hedgehog or Notch inhibitors, to improve outcomes underscores the resilience of CSCs and the importance of addressing their supportive environment (13). Hence, understanding CSC biology is crucial to overcoming PDAC drug resistance.

Beyond their inherent stemness, CSCs in PDAC exhibit remarkable plasticity, enabling them to interconvert with non-CSCs in response to microenvironmental cues. This phenotypic flexibility is driven by factors such as hypoxia, inflammatory cytokines, and ECM stiffness, which not only maintain CSC traits but also promote therapy evasion (14). For instance, hypoxia-induced HIF-1α activation enhances CSC stemness and triggers EMT, fostering both drug resistance and metastasis (15). Additionally, CSCs interact with tumor-associated macrophages (TAMs), cancer-associated fibroblasts (CAFs), and other stromal elements to maintain a protective niche (5, 11). TAM-derived cytokines such as TGF-β and IL-10 reinforce CSC survival, while CAFs contribute to ECM remodeling and metabolic crosstalk. This bidirectional interplay between CSCs and the tumor microenvironment (TME) creates a vicious cycle: CSCs remodel the TME to support their maintenance, and in return, the TME shields CSCs from immune and therapeutic attacks. These dynamic interactions complicate efforts to eliminate CSCs and explain the frequent relapses after initially successful therapies. Targeting CSCs in isolation may therefore be insufficient; effective interventions must disrupt the reciprocal CSC-TME dialogue.

This review aims to provide a comprehensive overview of CSCs in pancreatic cancer, with a particular focus on their mechanisms of immune evasion, therapy resistance, and plasticity. We explore the molecular features and key signaling pathways that sustain PaCSC stemness, the metabolic adaptations that confer survival advantages, and the intricate crosstalk between CSCs and various TME components such as CAFs, TAMs, and endothelial cells. Furthermore, we highlight emerging therapeutic strategies that target both CSCs and the stromal landscape to achieve durable treatment responses. By synthesizing current insights and recent clinical developments, this review seeks to identify opportunities for microenvironment-based reprogramming to synergistically disrupt CSC-driven disease progression in PDAC.

## 2 Features of PaCSCs

### 2.1 Biomarkers for PaCSCs

Since many markers expressed by CSCs are also found in tissue-resident stem cells and human embryonic stem cells, their characterization and isolation must rely on combinations of surface markers as well as both extracellular and intracellular markers. A comprehensive understanding of biomarkers beyond traditional surface markers will help better identify and isolate cells responsible for tumor initiation, drug resistance, and relapse.

CD44, CD133, CXCR4 and EpCAM are classic surface biomarkers for not only PaCSCs, but also other CSCs. The expression of CD44 is associated with increased proliferation, self-renewal and metastasis. For example, as few as a hundred CD44^high^/CD24^low/-^/Lineage^-^ breast cancer cells, could form tumors in mice, whereas tens of thousands of cells with alternate phenotypes failed to form tumors (16). The isoform of CD44, CD44v6, were found to be related with enhanced CSCs properties and resistance to anti-angiogenic therapies in PDAC (17). CD133 is a transmembrane glycoprotein belonging to Glycoprotein 1 family, and higher expression level of CD133 is closely associated with self-renewal and chemoresistance ability of PaCSCs. It is worthwhile to note that CD44^high^/CD133^high^ cells are found to exhibit increased tumorigenic capabilities across cancer types (18). EpCAM is a transmembrane glycoprotein predominantly expressed in epithelial cells and plays an essential role in regulating PaCSC survival and tumor growth (19).

Other biomarkers related to the metabolic state, CSC-specific signaling pathways and CSC niche regulation are critical for identifying and understanding the biology of PaCSCs. PaCSCs, like other CSCs, exhibit altered metabolism compared to differentiated tumor cells. Aldehyde Dehydrogenase 1 A1 (ALDH1A1) enables the metabolic reprogramming essential for PaCSC stemness and drug resistance by enhancing aerobic glycolysis, detoxifying reactive oxygen species (ROS), and increasing resistance to gemcitabine compared to normal PDAC cells (20). Additionally, PaCSCs upregulate Vimentin, N-cadherin, and transcription factors such as Snail and ZEB1 and gain enhanced capacity for EMT (21).

Previous studies have identified several markers for PaCSCs identification, including PIM-1, L1CAM, CD9, HNF1A, and DCLK1 (22–25). And recent studies have highlighted several novel markers for PaCSCs such as laminin γ2 (LAMC2) and Voltage-gated calcium channel α2γ1. A novel CSC subpopulation has been identified based on LAMC2 expression, which marks an aggressive signature in PaCSCs. This subpopulation is regulated by Smad/TGF-β pathways, and TGFBR1 inhibitors like vactosertib have shown to hinder PDAC progression (26). α2γ1 is involved in regulating various cellular processes, such as proliferation, survival, and migration, which are vital for the aggressive behavior of PaCSCs (27).

### 2.2 Key stemness signaling pathways

Several stemness pathways contribute to the maintenance and aggressive phenotypes of PaCSCs, enabling them to sustain their stem-like properties and negatively impact patients’ prognosis.

The Wnt/β-catenin signaling pathway is essential for maintaining the self-renewal and stemness of PaCSCs (28). This pathway regulates the translocation of β-catenin to the nucleus, where it activates the expression of genes that sustain the undifferentiated state of PaCSCs. Studies have shown that CD133^+^ PaCSCs rely on Wnt signaling to maintain their tumorigenic potential and resist differentiation (29). For instance, Wnt inhibitors such as XAV939 have been found to significantly reduce CSC-derived tumor growth, indicating the importance of this pathway in CSC maintenance and tumor initiation (30, 31).

Notch signaling is another key pathway involved in regulating PaCSC self-renewal and stemness. Activation of Notch receptors leads to the cleavage of the Notch intracellular domain, which translocates to the nucleus and activates the transcription of stemness-associated genes (32). In a pancreatic cancer model, Notch signaling was found to regulate the expansion of PaCSCs (33). Inhibiting Notch using GSI-18 (a γ-secretase inhibitor) significantly reduced PaCSC expansion and tumor growth, showing the essential role of Notch signaling in maintaining stemness and tumor initiation in pancreatic cancer (34–36).

Hedgehog signaling was found to be upregulated in PaCSCs, with the GLI1 transcription factor driving their tumorigenic potential (37). Hedgehog pathway inhibition using Vismodegib resulted in a significant reduction of PaCSC marker expression, tumor growth, and metastasis in animal models. This demonstrates the crucial role of Hedgehog signaling in supporting PaCSC self-renewal and metastatic spread (38).

**Figure 1:**
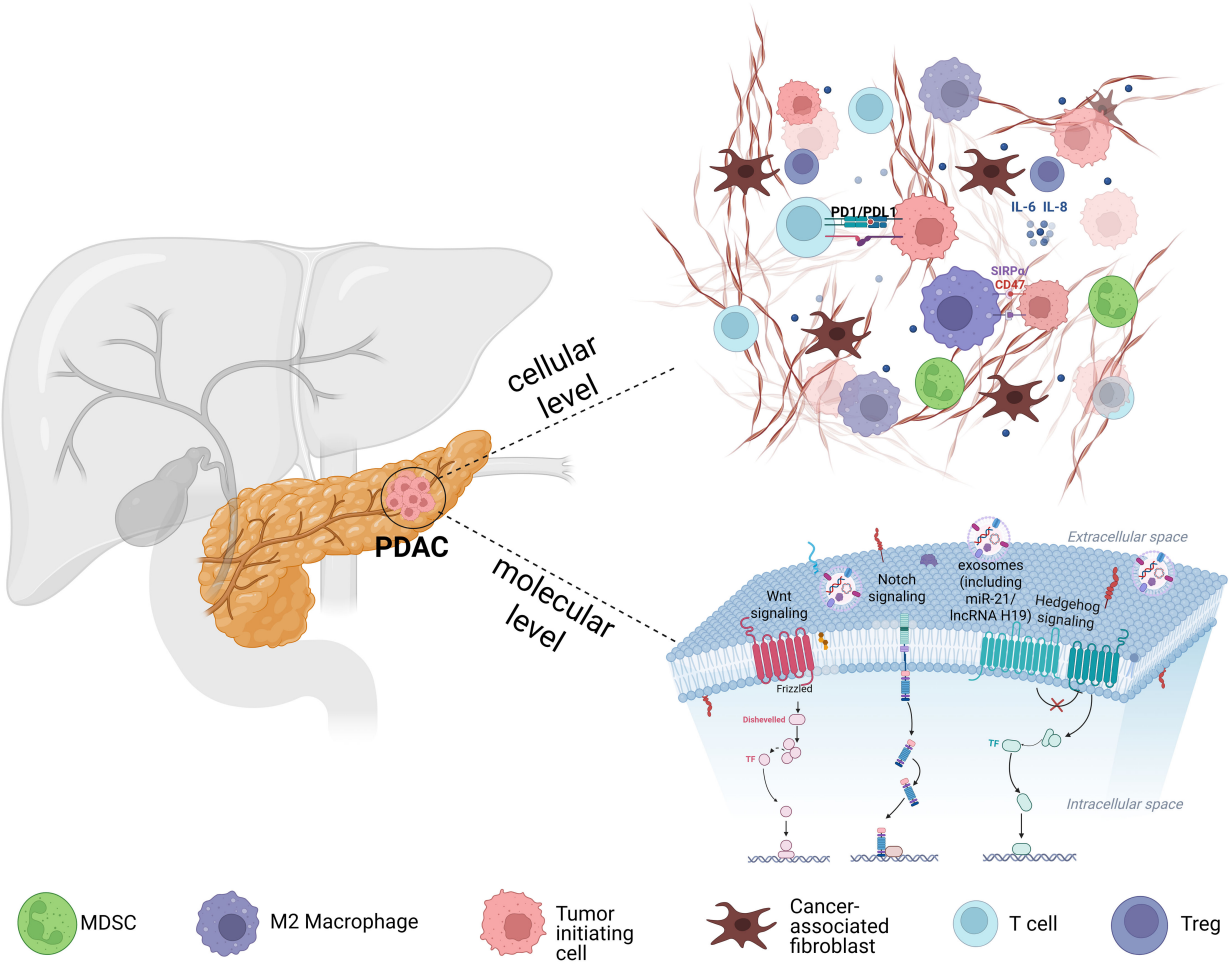
Schematic illustration of the PDAC microenvironment. The image depicts CAFs secrete cytokines such as IL-6 and TGF-β and remodel the ECM, while immunosuppressive cells (M2 macrophages, MDSCs, Tregs) further dampen T-cell infiltration and function, collectively reinforcing an immunosuppressive, therapy-resistant niche. Concurrently, CSCs sustaining their stemness and therapy resistance via developmental signaling pathways (Wnt, Notch, and Hedgehog) and dynamic chromatin states. CSCs also release exosomes containing regulatory molecules (e.g., PD-L1, miR-21, lncRNA H19) that promote immune evasion.

Additionally, TGF-β signaling regulates EMT, which enhances the migratory and invasive properties of PaCSCs, contributing to metastasis (14). TGF-β also helps PaCSCs survive under stress and evade treatment, promoting quiescence and therapy resistance (39). A study on PaCSCs in pancreatic cancer found that TGF-β signaling promoted EMT in these cells, enabling them to acquire invasive and metastatic capabilities (14). The inhibition of TGF-β signaling using SB431542 led to a decrease in PaCSC migration and metastasis, as well as an increase in chemotherapy sensitivity, highlighting the role of TGF-β in PaCSC metastasis and therapy resistance (40) (**Figure 1**).

### 2.3 CSC metabolic reprogramming

PaCSCs undergo metabolic reprogramming for stemness maintenance, survival in harsh microenvironments, and treatment resistance. One major aspect of this reprogramming involves glucose metabolism. PaCSCs exhibit dynamic metabolic flexibility, shifting between glycolysis and oxidative phosphorylation depending on the tumor ecosystem. During early tumorigenesis, PaCSCs upregulate aerobic glycolysis, an adaptation driven by the transcription factor MYC, which also enhances glutathione synthesis to manage oxidative stress. This metabolic switch enables PaCSCs to thrive in hypoxic niches and contributes to their proliferative and survival advantages (41). Furthermore, PaCSCs adapt their metabolic phenotype based on metastatic sites: for example, lung-metastatic cells favor OxPhos, while liver-metastatic ones rely more heavily on glycolysis. Lung-metastatic PaCSCs are enriched in ALDH^+^/CD133^+^ populations and exhibit a MET-like phenotype with oxidative metabolism (42). Liver-metastatic PaCSCs, on the other hand, display a drug-resistant, EMT-like phenotype, characterized by aerobic glycolysis and fatty acid β-oxidation (FAO)-mediated oxidative metabolism (42).

Antioxidant defense is another metabolic hallmark of PaCSCs. ALDH1A1, a recognized PaCSC marker, protects against oxidative stress, promotes stemness and contributes to gemcitabine resistance (43). Its activity is linked to enhanced ROS detoxification and survival in hypoxic or drug-exposed niches. Functional studies in ALDH1A1^+^ PaCSCs have shown elevated resistance to gemcitabine and greater tumorigenic potential compared to ALDH1A1^–^ counterparts (44).

As for amino acid and lipid metabolism, evidence remains limited and largely indirect. Some studies on PDAC (not specifically PaCSCs) have shown reliance on non-canonical glutamine metabolism and methionine cycling for NADPH production and epigenetic regulation (45, 46). While these pathways may be relevant to PaCSCs, their roles have not been directly validated in purified CSC populations and warrant further investigation.

### 2.4 CSC therapy resistance

PaCSCs exhibit robust chemotherapy resistance, particularly to gemcitabine, through several well-documented mechanisms. Upon gemcitabine treatment, there is a notable increase in CD24^+^ and CD133^+^ PaCSCs alongside upregulation of stemness-associated genes such as BMI1, Nanog, and SOX2. This shift is driven by a NADPH oxidase (NOX)-mediated ROS/NF-κB/STAT3 signaling cascade (47), which enhances phosphorylated STAT3 binding at promoters of these stemness genes, thereby boosting sphere formation, cell migration, chemoresistance, and tumor relapse (42, 43). Furthermore, functional studies show that CD133^+^ PaCSCs isolated from gemcitabine-resistant pancreatic cancer models demonstrate significantly greater resistance to gemcitabine compared to CD133^-^ cells (48). These cells also show enhanced 3D colony-forming ability and inherently higher tumorigenic potential (49).

In addition to chemoresistance, PaCSCs also display pronounced resistance to targeted therapies, largely due to activation of stemness-linked developmental pathways and their protective niche environments. For instance, Notch pathway activation sustains PaCSC survival and self-renewal despite conventional pathway inhibitors. In preclinical studies, inhibition of DLL4/Notch signaling with anti-DLL4 antibodies reduced the PaCSC fraction and partially restored drug sensitivity, but failed to fully eradicate PaCSCs without combination treatment (50). Apart from inhibitors of Notch signaling, inhibiting JNK signaling via MKP-1 induction by dexamethasone reduces surviving levels, increases gemcitabine sensitivity, and impairs PaCSC viability (51). Overall, single-agent targeted therapies often fall short against PaCSCs unless they directly impair self-renewal networks and are used along with conventional chemotherapy (52–55).

## 3 Interplay between PaCSCs and TME

### 3.1 Association between PaCSCs and hypoxia

**Figure 2:**
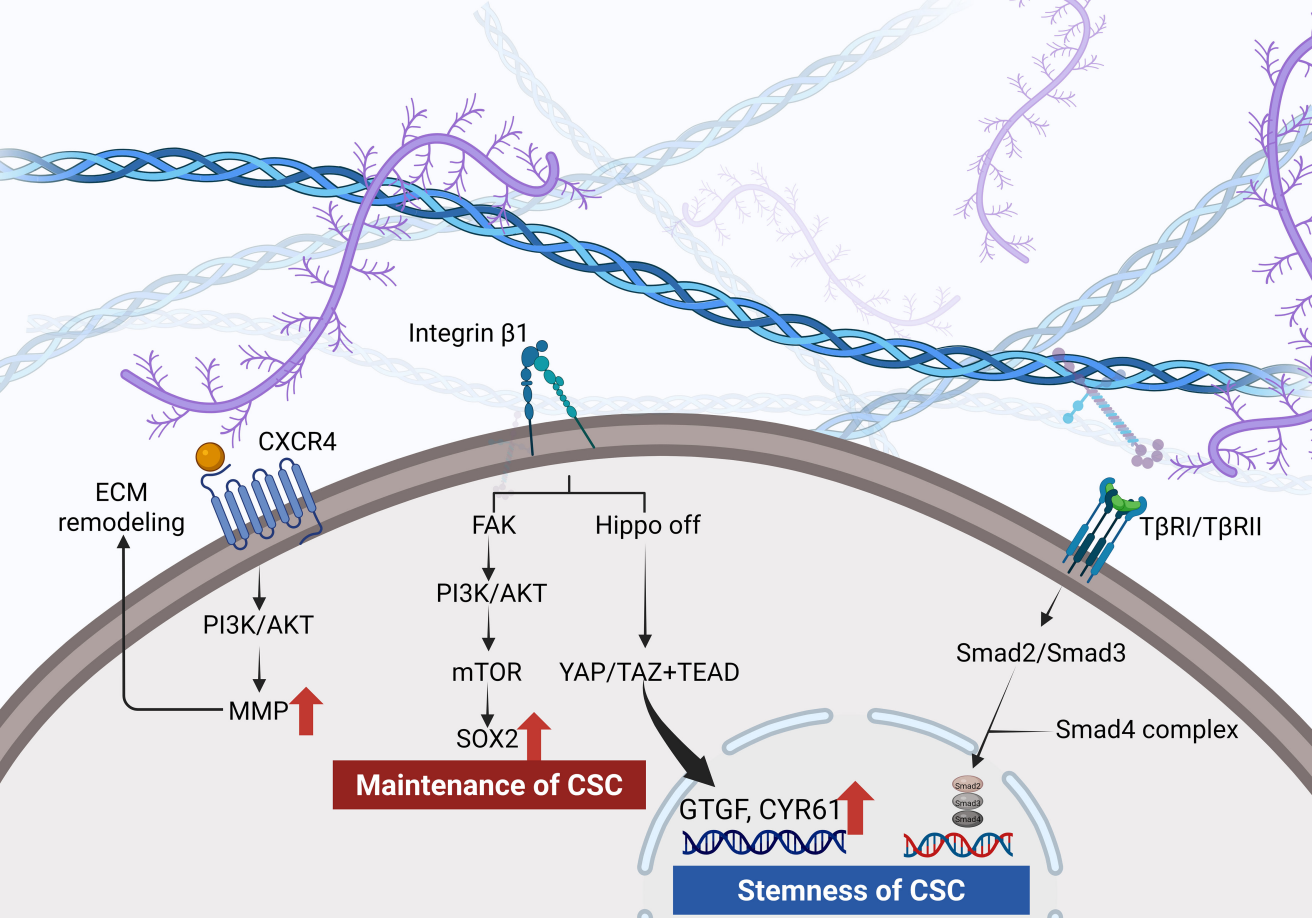
Hyaluronan engagement with CD44 activates PI3K/AKT and induces matrix metalloproteinase release. Integrin signaling through FAK drives a PI3K/AKT–mTOR–SOX2 axis that sustains CSC maintenance. Increased ECM stiffness inactivates the Hippo pathway, allowing YAP/TAZ and TEAD to upregulate CTGF and CYR61 and TGF−βreceptor signaling via Smad2, Smad3 and Smad4 promotes EMT and reinforces CSC stemness.

PaCSCs often exist in hypoxic niches within the TME, which serve as important regulators of their maintenance, plasticity, and aggressiveness. Hypoxia stabilizes HIF-1α, a key transcriptional regulator that activates pathways critical for stemness and invasion. For example, hypoxia activates the HIF-1α/Notch signaling axis, which promotes the expression of core stemness genes such as CD44, CD133, Nestin, and CXCR4, enhancing the dedifferentiation and tumorigenic potential of PaCSCs (56, 57) (**Figure 2**).

In addition, hypoxia induces autophagy in pancreatic cancer cells via HIF-1α signaling, facilitating both EMT and the conversion of non-CSCs into PaCSCs, further contributing to tumor dissemination (58) CD133^+^ PaCSCs themselves exhibit heightened expression of HIF-1α under hypoxia, directly linking this transcription factor to EMT progression and increased migratory capacity (59). Moreover, hypoxic cancer cells release exosomes enriched with lncRNAs such as lncROR, which inactivate the Hippo-YAP pathway in recipient PaCSCs and thereby enhance chemoresistance and stem-like features (60).

### 3.2 Association between PaCSCs and angiogenesis

PaCSCs orchestrate angiogenesis through multiple mechanisms, underlining their role in shaping the tumor vascular niche and contributing to therapeutic resistance. One key mechanism involves the activation of STAT3 signaling (14, 61). STAT3 activation in pancreatic cancer cells, including those with stem-like traits, directly binds to the VEGF promoter, upregulates VEGF expression, and enhances endothelial cell proliferation and migration, leading to increased angiogenesis and tumor growth *in vivo* (62). Further mechanistic studies demonstrate that STAT3 cooperates with Sp1 and HIF-1α at the VEGF promoter to amplify expression of angiogenic factors such as VEGF and bFGF (63). PaCSCs also influence the vascular niche through recruitment and activation of stromal cells. TAMs and mesenchymal stem cells (MSCs), often within CSC niches, secrete VEGF-A and PDGF, augmenting endothelial proliferation and vessel formation (64).

### 3.3 Association between PaCSCs and TAMs

TAMs play a crucial role in supporting PaCSCs by promoting stemness, therapy resistance, and immune evasion. For example, the coexistence of CD44^+^/CD133^+^ PaCSCs with CD204^+^ TAMs is associated with worse prognosis, indicating that macrophage infiltration supports the CSC niche and contributes to poor outcomes. Additionally, TAM-derived CD51 enhances PaCSC properties by activating the TGF-β1/Smad2/3 signaling axis, leading to increased self-renewal and tumorigenic potential (65). Similarly, exosomes derived from M2-polarized macrophages deliver microRNA-21-5p to PaCSCs, promoting their differentiation and activity by targeting KLF3, a known tumor suppressor (66).

TAMs also contribute to chemotherapy resistance. After chemotherapy-induced apoptosis, macrophages polarize toward an M2 phenotype and secrete factors such as 14-3-3ζ that enhance PaCSC resistance to drugs like gemcitabine and abraxane. These chemoresistant cells show increased sphere formation and tumorigenesis, confirming enrichment in CSCs (67).

### 3.4 Association between PaCSCs and CAFs/ECM

PaCSCs interact intricately with CAFs and ECM, shaping a TME that sustains tumorigenesis, therapy resistance, and immune evasion. CAFs are key players in forming the ECM that supports PaCSC stemness and survival. This matrix is often assembled via fibronectin-binding integrins (α5β1 and αvβ3) on CAFs, and targeting these integrins can disrupt ECM assembly and reduce stemness of PaCSCs (68). CAFs also secrete various ECM proteins and remodeling enzymes that reinforce ECM stiffness and provide biochemical signals to PaCSCs, promoting their resistance to chemotherapeutics and enhancing their metastatic potential (69).

Beyond mechanical support, CAFs contribute to metabolic reprogramming in the TME, which benefits PaCSCs by providing alternative nutrients and promoting immune evasion (70, 71). Furthermore, CAF-derived ECM components such as fibronectin and laminin trigger NADPH oxidase mediated ROS production in PaCSCs, enhancing their survival through prosurvival pathways including 5-lipoxygenase signaling (72).

It is important to note that the structural and functional heterogeneity of CAFs means that some subtypes may suppress, while others promote, tumor progression. Specifically, the TME comprises three major subtypes of CAFs, namely myofibroblastic CAFs (myCAFs), inflammatory CAFs (iCAFs), and antigen-presenting CAFs (apCAFs), each with unique spatial localization and functional roles (73–75). These insights indicate that simply eliminate CAF population may hinder treatment efficacy.

### 3.5 The association between PaCSCs and exosomes

PaCSCs actively engage in bidirectional communication with components of the TME through exosomes, thereby driving tumor progression, modulating immune responses, and promoting therapeutic resistance. PaCSC-derived exosomes are key vehicles for horizontal gene transfer and intercellular communication. They carry proteins, mRNAs, miRNAs, and other bioactive molecules that influence stromal and immune cells in the TME (76). PaCSC-secreted exosomes have been found to contain CD133, a well-known CSC marker, indicating their role in maintaining stemness and potentially predicting chemoresistance in advanced pancreatic cancer (77). Furthermore, bone marrow mesenchymal stem cell (BM-MSC)-derived exosomes can suppress PaCSC stemness by delivering circular RNAs such as circ_0030167, which inhibits the Wnt/β-catenin signaling pathway (78).

Although research on the roles of exosomes in PaCSCs remains limited, their functions have been more extensively characterized in other cancer types, providing valuable insights into their likely relevance in PaCSCs. For example, in breast cancer, CSC-derived exosomes enriched with miR-21 and miR-155 have been shown to induce fibroblast transformation into CAFs, promoting tumor invasion and chemoresistance (79). In thyroid cancer, hypoxic CSCs secrete exosomes containing LINC00665, which enhances EMT and stemness traits in recipient tumor cells, thereby accelerating metastasis (80). In prostate cancer, exosomes from CSCs can reprogram MSCs into myofibroblast-like cells that facilitate angiogenesis and tumor growth (81). Moreover, in colorectal cancer, CSC-derived exosomes have been shown to deliver Wnt signaling components to neighboring cells, sustaining a pro-stemness microenvironment and enhancing metastatic capability (82). These findings across tumor types collectively suggest that CSC-derived exosomes are powerful mediators of intercellular communication and could play similar roles in PaCSCs, such as contributing to immune suppression, therapeutic resistance, and metastatic potential. Future studies focusing on PaCSC-specific exosomal profiles and their functional roles are essential to develop novel diagnostic markers and targeted therapies.

## 4 Targeted therapy

### 4.1 Targeting CSC biomarkers

CSC-specific surface antigens and epigenetic vulnerabilities are leveraged for precision therapies. CAR-T/NK therapies targeting Claudin18.2 and CD133 demonstrate clinical promise: Claudin18.2-directed CAR-T cells reduce ALDH1A1^+^ CSCs in metastatic PDAC (83), while CD133-targeted CAR-NK cells suppress metastasis in preclinical models (84). To overcome stromal barriers, these therapies are combined with hyaluronidase or FAK inhibitors, enhancing intratumoral penetration and survival (85, 86). Epigenetic approaches include EZH2 inhibitors that force CSC differentiation by silencing stemness genes, and HAD inhibitors that activate STING-dependent immunity via viral mimicry (87, 88). Besides, antibody–drug conjugates and bispecific T-cell engagers (BiTEs) against CD133 or CD44 are under preclinical, with anti-CD133 ADCs and CD133^+^CD3^+^ BiTEs showing efficient CSC killing and sphere‐formation blockade (89, 90). Radioimmunotherapy against tuft-cell marker DCLK1 is being explored to deliver localized radiation to CSC niches, further illustrating the therapeutic promise of biomarker-guided approaches (91). Furthermore, Anti-CD47 antibody therapy disrupts the signal on CD34^+^CD38^-^ leukemic stem cells, enhancing macrophage-mediated phagocytosis and achieving durable remissions in preclinical Acute Myeloid Leukemia (AML) models (92, 93). Collectively, these strategies exploit PDAC and other malicious tumor CSC vulnerabilities to improve eradication of the cells driving tumor recurrence and therapy resistance.

### 4.2 Targeting key signaling pathways

Critical pathways driving CSC-stroma crosstalk are pharmacologically targeted. Wnt/β-catenin and Hedgehog inhibitors block desmoplasia and CSC stemness but face adaptive resistance via TIM-3 upregulation, necessitating combination with dual checkpoint inhibitors (anti-CD47 plus anti-PD-1) to enhance phagocytosis and T-cell cytotoxicity (92, 94, 95). Hypoxia-HIF-1α targeting (PX-478) and YAP/TAZ inhibition reverse metabolic reprogramming and ECM-driven chemoresistance, with verteporfin plus losartan reducing metastases (96, 97). Metabolic-immune synergy is exemplified by MCT1 inhibitors combined with anti-CTLA-4, normalizing extracellular pH and reversing lactate-driven immunosuppression, enhancing survival time (98). Emerging data indicate that MLKL-mediated necroptosis in PDAC fosters an immunosuppressive niche by recruiting TAMs and upregulating the CD47 signal, while necroptosis-induced macrophage extracellular traps degrade the ECM and activate CXCL8 to drive EMT and ICAM-1–dependent endothelial adhesion. Dual inhibition of MLKL and CD47 in preclinical PDAC models markedly reduces liver metastases (99).

## 5 Conclusion

PaCSCs play a central role in therapy resistance, metastasis, and immune evasion in PDAC. Their dynamic interaction with TME, including CAFs, TAMs, hypoxia, and ECM, creates a protective niche that reinforces stemness and limits treatment efficacy. Monotherapies targeting single pathways have largely failed due to compensatory mechanisms and the plasticity of CSCs. Emerging multimodal strategies that simultaneously target CSCs and reprogram the TME show greater promise. Approaches such as CAR-T/NK cell therapies, metabolic inhibitors, and epigenetic modulators offer new avenues for durable responses. Future efforts should integrate spatial omics and biomarker-guided precision to optimize treatment timing and sequencing, ultimately transforming the management of this aggressive malignancy.
